# Assessment of mesh displacement following laparoscopic enhanced view totally extraperitoneal technique: comparing mesh fixation and non-fixation in difficult inguinal hernias

**DOI:** 10.1007/s13304-025-02271-5

**Published:** 2025-05-28

**Authors:** Abdullah Hilmi Yilmaz, Mehmet Esref Ulutas

**Affiliations:** 1 Department of Surgery, Van Training and Research Hospital, University of Health Sciences, Süphan Mahallesi Hava Yolu Kavşağı 1. Kilometre EDREMIT/Van, Van, Türkiye; 2 Department of Surgery, Gaziantep City Hospital, University of Health Sciences, Gaziantep, Türkiye

**Keywords:** Mesh displacement, Mesh fixation, Difficult cases, ETEP

## Abstract

**Background:**

Mesh fixation, one of the controversial issues of laparoscopic surgery, has not been investigated in the enhanced view-totally extraperitoneal (eTEP) technique. This study aimed to compare mesh fixation (FX) and non-fixation (NFX) in difficult cases in the eTEP technique.

**Methods:**

A total of 61 consecutive patients diagnosed with inguinal hernias who underwent the eTEP procedure were randomized for the study. The groups were FX and NFX. The primary outcome was mesh displacement; secondary outcomes were recurrence, return to normal life, pain, and postoperative findings.

**Results:**

Mesh displacement, recurrence, mean operative time, length of stay, pneumoperitoneum, and postoperative complications were similar. VAS scores were higher in the FX group (*p* = 0.017, *p* < 0.001, *p* < 0.001, respectively). Return to normal life was more days in the FX group (*p* = 0.02). The mesh displacement at 6 months was higher than at 1 month in both groups (*p* < 0.001).

**Conclusions:**

There is no increased risk of mesh displacement and recurrence in difficult cases, such as scrotal and recurrent hernias, without mesh fixation. With the eTEP technique, mesh fixation can be safely avoided for less pain and a faster return to normal life.

Trial registration: Clinicaltrials number: NCT06417359.

## Introduction

Inguinal hernia surgery represents one of the most prevalent procedures within the field of general surgery. While laparoscopic techniques typically present a steeper learning curve compared to open surgical techniques, they offer several advantages, including expedited recovery times and reduced rates of both acute and chronic pain [1]. According to the updated guidelines from the International Herniasurge, the utilization of laparoscopic techniques is advised for patients with primary unilateral inguinal hernia, provided that a specialized hernia surgeon is available and possesses the necessary resources. This recommendation is based on the lower rates of postoperative pain and the reduced occurrence of chronic pain associated with this surgical approach [2].

The totally extraperitoneal (TEP) and transabdominal preperitoneal (TAPP) techniques are the leading laparoscopic approaches for inguinal hernia repairs. The enhanced view-totally extraperitoneal (eTEP) technique represents a modified approach to the traditional TEP technique. This method, characterized by an expanded field of view and distinct trocar access, was introduced by J. Daes in 2012. Daes noted that the eTEP technique proves particularly advantageous in challenging cases, including scrotal hernias, bilateral hernias, incarcerated hernias, instances involving patients with obesity, and cases where there exists a limited distance between the navel and the pubic bone [3]. This technique has a large extraperitoneal space, so accidental pneumoperitoneum can be easily tolerated. 

The debate surrounding mesh fixation (FX) in laparoscopic techniques is highly relevant and continues to be at the forefront of surgical discussions. In the TEP technique, mesh FX is not recommended in the international guidelines of the Herniasurge group [1]. Nevertheless, tackers, tissue adhesives, or suturing the mesh are the most common methods for FX. Tissue adhesives are costly and sometimes not preferred because they cause allergic reactions [1, 4]. Several studies have indicated that the recurrence of conditions may arise due to insufficient fixation of the mesh or the failure to secure the mesh appropriately [5, 6]. A meta-analysis shows that hernia recurrence is not associated with mesh FX [7]. However, several studies clearly demonstrate that mesh FX can increase the likelihood of postoperative chronic pain [8, 9].

The eTEP technique is an invaluable and highly effective approach to managing difficult cases. There is no current information about mesh fixation in these difficult cases using the eTEP technique. Based on this, we wanted to evaluate the effect of mesh FX or non-fixation (NFX) on mesh displacement in scrotal hernias and recurrent hernias in the eTEP technique. The objective of this study was to conduct a comparative analysis of mesh FX and NFX in challenging cases utilizing the eTEP technique. This study is the first of its kind in the literature to examine mesh FX and NFX within the eTEP technique.

## Materials and methods

**Fig. 1:**
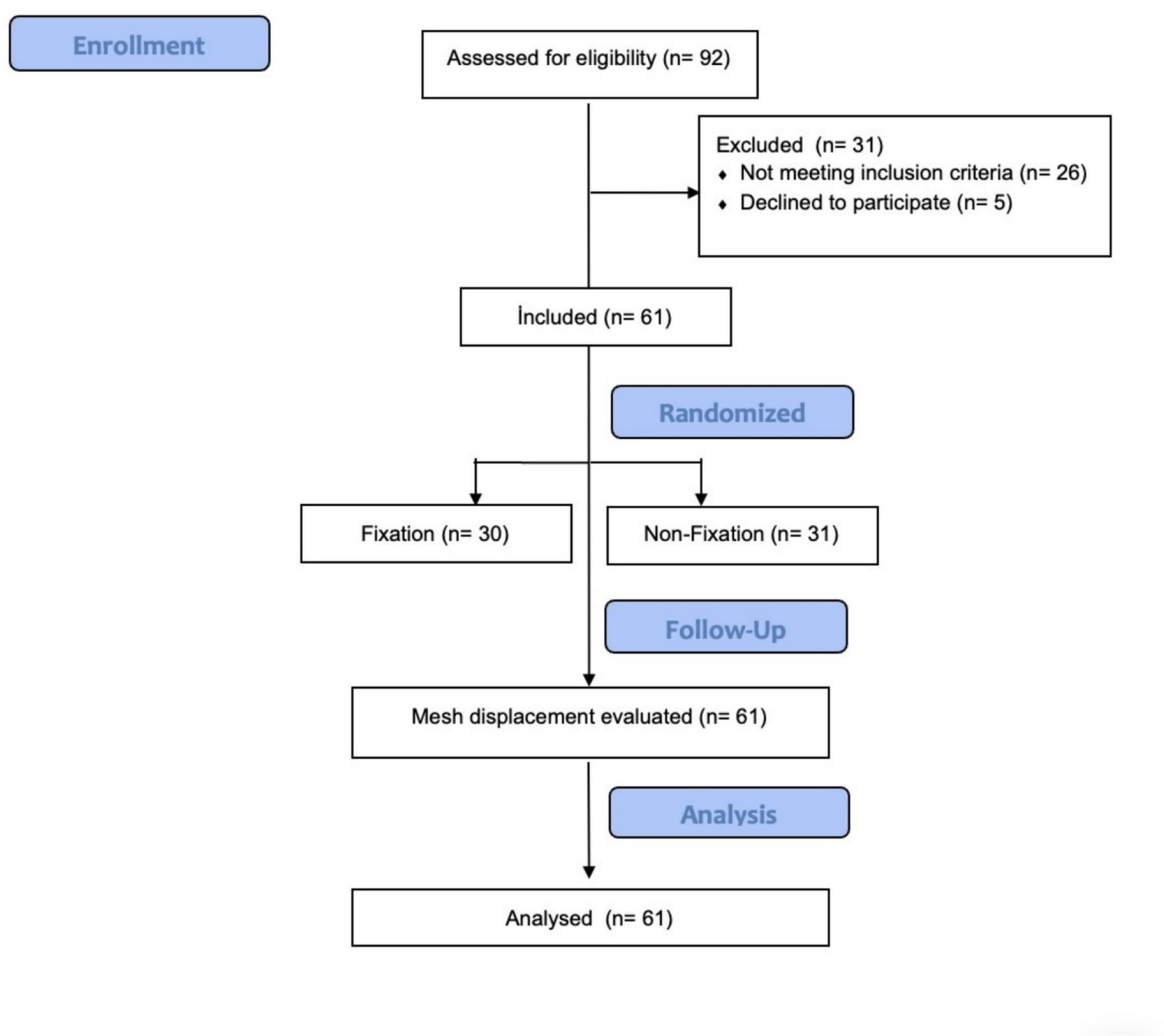
Flow Diagram

Between November 2023 and February 2024, 61 patients with unilateral inguinal hernia were randomized into two groups: 30 in the Fixation (FX) group who had eTEP with mesh fixation, and 31 in the non-fixation (NFX) group who had eTEP without mesh fixation. The flow diagram is shown in Fig. 1.

Laparoscopic inguinal hernia repairs were performed by a highly qualified surgeon with specialization in this technique, who has utilized the eTEP approach in over 200 procedures. The study employed a double-blind design, whereby both the participants and the researcher responsible for conducting measurements and documenting parameters during the patients'follow-up were unaware of the specific study groups to which the patients were assigned.

Patients aged 18–65, unilateral recurrent inguinal hernias, and scrotal hernias were included. Individuals who are under 18 years of age or over 65 years of age, as well as those presenting with incarcerated inguinal hernias, bilateral inguinal hernias, and large direct hernias, were excluded from this study.

This prospective randomized study has been undertaken in collaboration with the Department of Surgery, Van Training and Research Hospital, University of Health Sciences. The study protocol was compliant with CONSORT 2010 instructions and registered on ClinicalTrials.gov (NCT06417359).

### Sample size

Based on Cohen’s power analysis, which was conducted using expert guidance, it was determined that to achieve a statistical power of 80% with a large effect size of 0.80 and a significance level (alpha) of 0.05, fifty-two patients should be included, with 26 patients in each group. In consideration of a projected patient attrition rate of 10%, 30 patients were enrolled per group, resulting in a total of 60 patients for the study.

### Randomization

All 61 patients were randomly assigned into two groups using a computed randomization sequence (1:1).

### Surgical methods

**Fig. 2:**
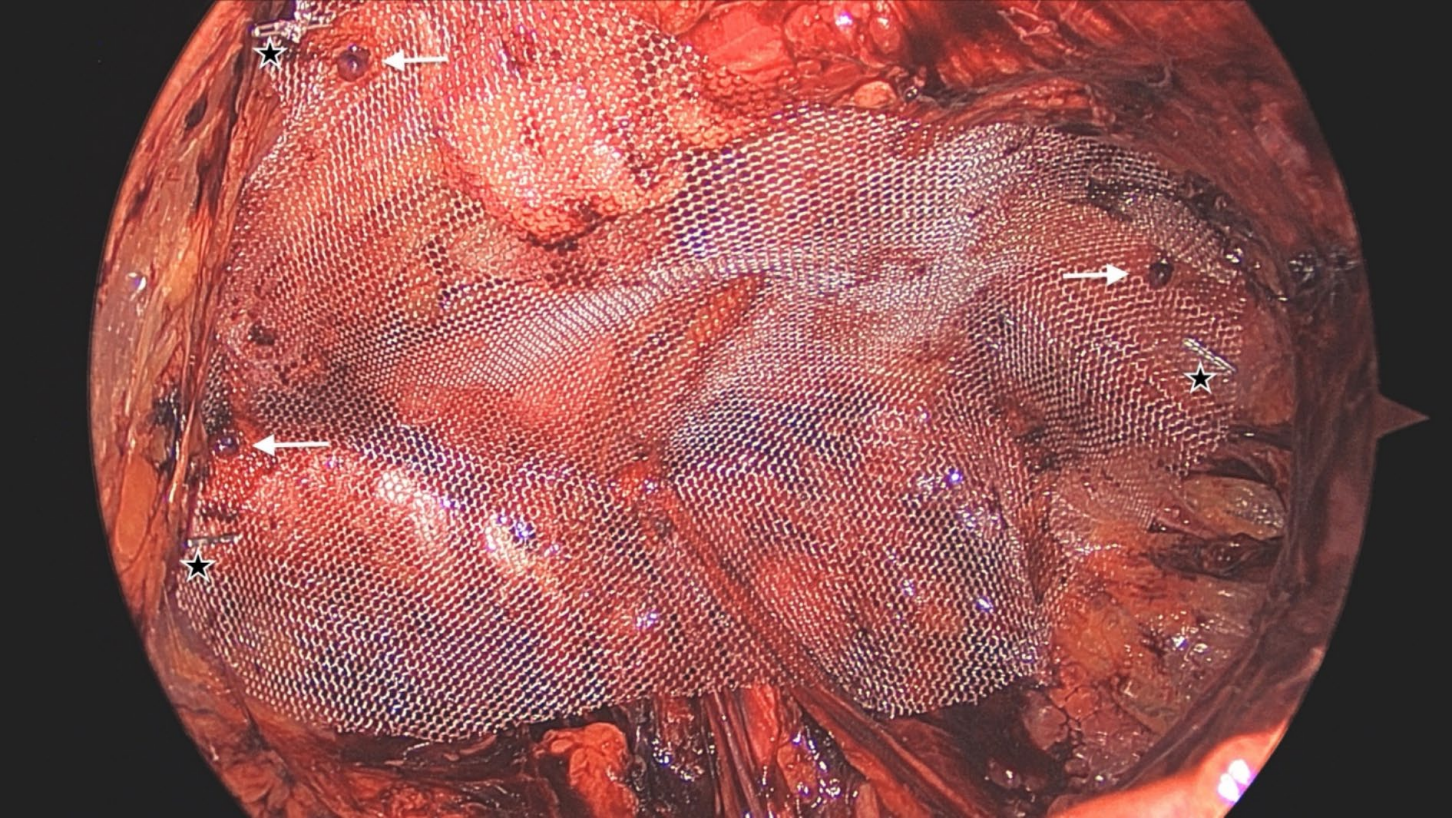
Mesh placement (stars mark clips, arrows mark tackers)

All patients underwent surgical procedures under general anesthesia and were administered an intravenous infusion of 1 g of cefazolin preoperatively. To ensure adequate postoperative analgesia, a routine intravenous infusion of 1 g of paracetamol and 1–2 mg/kg of tramadol was provided to each patient. Each individual was subsequently placed on a polypropylene mesh measuring 15 × 12 × 10 cm, with the superomedial, inferomedial, and lateral regions indicated by the placement of 10 mm clips (Fig. 2, stars mark clips).

#### eTEP procedure

After establishing a sterile field, an incision was performed 5 cm superolateral to the navel, on the side opposite to the hernia. Following the incision of the skin, the rectus sheath was clearly visualized and subsequently incised. The fibers of the rectus muscle were meticulously separated, allowing for visualization of the posterior rectus sheath (PRS). A 10 mm primary trocar was positioned on the PRS. Insufflation was conducted at a pressure of 10 mmHg, and blunt dissection with a telescope commenced. Subsequently, a 5 mm secondary trocar was inserted at the junction of the semilunar line and the arcuate line on the opposite side of the hernia. The retrorectus region, particularly in proximity to the midline, was meticulously dissected utilizing an energy device. The PRS was transected under direct visualization with scissors. A third 5 mm trocar was introduced into the extraperitoneal space via the umbilicus. Following the insertion of trocars, the procedure to establish a critical view of the myopectineal orifice (CV-MPO) was successfully executed [10]. Preperitoneal dissection was performed medially, extending to a depth of 3–4 cm inferior to the pubic bone, and laterally within the Bogros cavity until the psoas muscle was visualized. The hernia was meticulously dissected, and in cases of recurrent hernias, the peritoneal sac was thoroughly reduced. In scrotal hernias, the hernia sac was dissected from the cord, its elements, and testicular vessels. The hernia sac was first opened in the middle part, and it was seen that there was no structure inside, and the hernia sac was transected. The proximal hernia sac was tied. The distal sac was left. The peritoneal loop [11] was divided. The iliac vessels, cord, structures, and psoas muscle were better visualized. The designated polypropylene mesh was positioned to ensure an overlap of at least 3–4 cm over the sites of direct, indirect, and femoral hernias, with an additional margin of 2–3 cm below the pubic region. In the FX group, the mesh was fixed in 3 places: Cooper ligament, lateral, and superomedial area. Absorbable tackers were used (Fig. 2, arrows mark tackers). The mesh was not fixed in the NFX group. During the desufflation process conducted under direct visualization, careful measures were implemented to prevent both mesh displacement and crimping of the mesh.

### Radiological evaluation

**Fig. 3:**
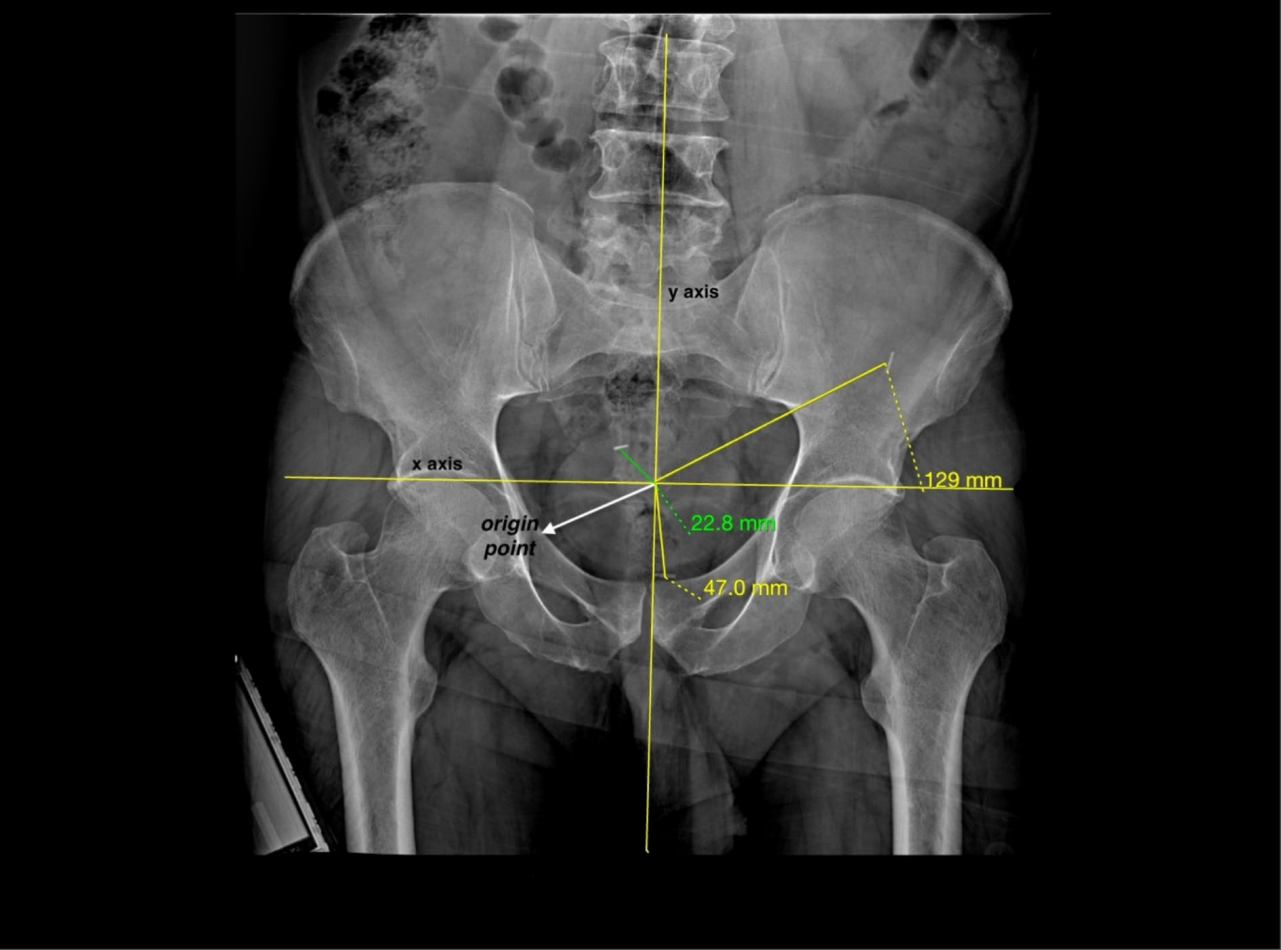
Postoperative radiography

Pelvic radiography was performed on the first postoperative day, in addition to evaluations conducted 1 month and 6 months after the surgical procedure. The radiologist was unaware of the specific surgical procedures performed on the patients and was blinded to this information. The attachments on the mesh are designated as superomedial clip 1, inferomedial clip 2, and lateral clip 3. The intersection of the line connecting the lumbar vertebral processes and the symphysis pubis, represented along the vertical axis (y-axis), with the tangent line intersecting the right and left acetabulum along the horizontal axis (x-axis), is referred to as the point of origin. The distances of the clips were measured relative to their point of origin, as illustrated in Fig. 3. The postoperative radiographic images and corresponding measurements of the clips are presented in this figure. The displacements of the clips, observed between the second and third radiographs in comparison to the first, are designated as “d.”. Total mesh displacement was calculated by summing the displacement of clips 1, 2, and 3.

### Outcomes

All clinical data, including demographic characteristics such as age, sex, and body mass index of the patients, were meticulously recorded. Additional information documented included the duration of the operation (from skin to skin), types of inguinal hernias, presence of pneumoperitoneum, postoperative complications, length of hospitalization, and postoperative pain scores measured on the first day using a numerical scale. Patients were scheduled for follow-up evaluations at 1 month and 6 months postoperatively. The patients’ numerical pain scores and days to return to normal life were also recorded. Return to normal life is defined as returning to work for the employed person and doing all household chores without any assistance from the person at home.

This study’s primary outcome was mesh displacement; the secondary outcomes of the study included the recurrence of hernia, postoperative complications, and levels of pain experienced by patients.

### Statistical analyses

Prior to conducting further analyses, the Kolmogorov–Smirnov tests were employed to evaluate the normality of the variables under consideration. Non-parametric methods were utilized for variables that did not conform to a normal distribution. Subsequently, the Mann–Whitney U test was employed to compare measurements obtained between groups. The relationships or differences between groups concerning categorical variables were analyzed using Chi-square and Fisher’s exact tests. For multiple group comparisons, an analysis of variance (ANOVA) was implemented. Depending on the homogeneity of variances, either the Bonferroni or Tamhane-T2 tests were applied. Repeated Measures ANOVA was utilized to analyze repeated measurements. The comparative results between groups with respect to other demographic characteristics were presented as ratios of qualitative variables, while quantitative variables were expressed as means and standard deviations. Statistical analyses were carried out using the Statistical Package for the Social Sciences (SPSS), version 22.0 (SPSS Inc., Chicago, IL, USA), with a p-value of less than 0.05 deemed statistically significant across all analyses.

## Results

Of the 61 eligible patients, 30 were randomized to the FX group and 31 to the NFX group. All patients were male. The mean age of the FX and NFX groups was 42.7 (± 10.5) and 43.9 (± 14.6). In the FX group, there were ASA 1 10 (33.3%), ASA 2 18 (60%), ASA 3 2 (6.7%) patients; in the NFX group, there were ASA 1 14 (45.2%), ASA 2 11 (48.4%), ASA 3 2 (6.5%) patients. Indirect inguinal hernia was 19 (63.3%) in the FX group and 22 (71%) in the NFX group. Direct inguinal hernia was 9 (30%) in the FX group and 6 (19.4%) in the NFX group. Femoral hernia was 1 (3.3%) in the FX group and 2 (6.5%) in the NFX group. The presence of lipoma was 1 (3.3%) in both groups. Scrotal hernia was 15 in both groups. Recurrent hernia was 15 (50%) in the FX group and 16 (51.6%) NFX group. The mean BMI of the FX group was 26.6 (± 3.3), and the NFX group was 26.6 (± 3.5). Demographic characteristics and operative and postoperative data of the patients are shown in Table 1.

**Table 1:** Demographic characteristics of the patients and operative and postoperative data

	Fixation (*n* = 30)	Non-fixation (*n* = 31)	*p*
Age	42.7 ± 10.5	43.9 ± 14.6	0.7
Male/Female	30/0	31/0	–
ASA			0.63
I	10 (% 33.3)	14 (% 45.2)	
II	18 (% 60)	15 (% 48.4)	
III	2 (% 6.7)	2 (% 6.5)	
HC			0.77
Indirect	19 (% 63.3)	22 (% 71)	
Direct	9 (% 30)	6 (% 19.4)	
Femoral	1 (% 3.3)	2 (% 6.5)	
Lipoma	1 (% 3.3)	1 (% 3.2)	
Hernia Type			0.55
Scrotal	15 (% 50)	15 (% 48.4)	
Recurrent	15 (% 50)	16 (% 51.6)	
BMI	26.6 ± 3.3	26.6 ± 3.5	0.97
Complication			0.94
None	18 (% 60)	18 (% 58.1)	
Hematoma	5 (% 16.7)	7 (% 22.6)	
Seroma	6 (% 20)	5 (% 16.1)	
SSI	1 (% 3.3)	1 (% 3.2)	
Op. time(min.)	43 ± 8.1	43.8 ± 9.9	0.75
LOS (day)	1	1	–
PP	25 (% 83.3)	25 (% 80.6)	0.57
Return to normal life (days)	9.2 ± 2.8	7.6 ± 2.7	**0.02**
Recurrence	0	0	–

*ASA* American society of anaesthesiologists, *HC* hernia classification, *Op*. operation, *BMI* body mass index, *LOS* lenght of stay, *SSI* surgical site ınfection, *PP* pneumoperitoneum, Significance level is p=0.05

The mean operative time was 43 (± 8.1) in the FX group and 43.8 (± 9.9) in the NFX group, and there was no statistical difference (*p* = 0.75). The length of stay was 1 day in both groups. Pneumoperitoneum was 25 (83.3%) in the FX group and 25 (80.6%) in the NFX group (*p* = 0.57). Hematoma was 5 (16.7%) in the FX group and 7 (22.6%) in the NFX group. Seroma was 6 (20%) in the FX group and 5 (16.1%) in the NFX group. SSI was 1 in both groups. The mean return to normal life was 9.2 (± 2.8) days in the FX group and 7.6 (± 2.7) days in the NFX group (*p* = 0.02). A follow-up of at least 6 months was performed for all patients. No recurrence was observed in any patient in either group.

### Mesh displacement

There was no statistical difference between the FX and NFX groups in the displacement of each clip and total mesh displacement (Table 2). There was no statistical difference in total mesh displacement between the FX and NFX in scrotal and recurrent hernia subgroups (Table 3). In the scrotal hernia subgroup in the NFX group, the 1 st clip was displaced more than the other clips at 1 month and 6 months (*p* = 0.02, *p* = 0.01). Except for this information, all clips were displaced at a similar rate when compared with each other (Table 4). The mesh displacement observed at 6 months was greater than that recorded at 1 month in both groups (*p* < 0.001, Table 5).

**Table 2:** Mesh displacement

		Fixation	Non-Fixation	*p*
Month 1	Clip 1 d	0.8 (± 0.5)	0.8 (± 0.5)	0.75
	Clip 2 d	0.6 (± 0.4)	0.6 (± 0.4)	0.97
	Clip 3 d	0.8 (± 0.5)	0.6 (± 0.4)	0.20
	Total d	2.1 (± 1)	2 (± 0.8)	0.98
Month 6	Clip 1 d	0.9 (± 0.5)	1 (± 0.5)	0.51
	Clip 2 d	0.8 (± 0.3)	0.6 (± 0.3)	0.11
	Clip 3 d	0.9 (± 0.6)	0.7 (± 0.5)	0.26
	Total d	2.5 (± 1)	2.3 (± 0.8)	0.40

Total d = Clip 1 + 2 + 3 d, d: displacement (cm)

**Table 3:** Total mesh displacement in subgroups

		Fixation	Non-Fixation	*p*
Scrotal Hernia	1.Month Total d	2.1 (± 1)	1.9 (± 0.7)	0.77
	6.Month Total d	2.5 (± 1)	2.2 (± 0.4)	0.37
Recurrent Hernia	1.Month Total d	2.1 (± 1.1)	2.1 (± 1)	0.74
	6.Month Total d	2.5 (± 1)	2.4 (± 1)	0.63

Total d = Clip 1 + 2 + 3 d, d: displacement (cm)

**Table 4:** Clips movement

			Clip 1 d	Clip 2 d	Clip 3 d	*p*
Fixation	Scrotal Hernia	1. Month	0.8 (± 0.4)	0.6 (± 0.4)	0.7 (± 0.5)	0.16
		6. Month	0.8 (± 0.5)	0.7 (± 0.3)	1 (± 0.6)	0.25
	Recurrent Hernia	1. Month	0.8 (± 0.6)	0.6 (± 0.4)	0.8 (± 0.5)	0.79
		6. Month	1 (± 0.5)	0.8 (± 0.3)	0.8 (± 0.5)	0.30
Non-Fixation	Scrotal Hernia	1. Month	0.9 (± 0.4)*	0.5 (± 0.4)	0.5 (± 0.3)	**0.02**
		6. Month	1.1 (± 0.4)*	0.5 (± 0.3)	0.6 (± 0.3)	**0.01**
	Recurrent Hernia	1. Month	0.8 (± 0.6)	0.6 (± 0.3)	0.7 (± 0.4)	0.54
		6. Month	0.9 (± 0.5)	0.7 (± 0.3)	0.8 (± 0.7)	0.49

Each superscript (*) indicates a statistically significant difference from other subgroups at p < 0.05. d: displacement (cm), Significance level is p=0.05

**Table 5:** Total mesh displacement by months

		1. Month Total d	6. Month Total d	*p*
Fixation	Scrotal Hernia	2.1 (± 1)	2.5 (± 1)	** < 0.001**
	Recurrent Hernia	2.1 (± 1.1)	2.6 (± 1)	** < 0.001**
Non-Fixation	Scrotal Hernia	1.9 (± 0.7)	2.2 (± 0.4)	** < 0.001**
	Recurrent Hernia	2.1 (± 1)	2.4 (± 1)	** < 0.001**

Total d = Clip 1 + 2 + 3 d, d: displacement (cm), Significance level is p=0.05

### VAS scores

In all patients, VAS scores on day 1, month 1, and month 6 were higher in the FX group. (*p* = 0.017, *p* < 0.001, *p* < 0.001, respectively, Table 6).

**Table 6:** VAS scores

		Fixation	Non-Fixation	p
VAS 1. Day	All Patients	4.1 (± 1.1)	3.4 (± 1)	**0.017**
	Scrotal	3.8 (± 1.1)	3.4 (± 1)	0.31
	Recurrent	4.4 (± 1)	3.5 (± 1)	**0.016**
VAS 1. Month	All Patients	2.7 (± 1.1)	1.7 (± 1)	** < 0.001**
	Scrotal	2.9 (± 0.9)	1.9 (± 0.9)	**0.006**
	Recurrent	2.5 (± 1.3)	1.5 (± 1.1)	**0.037**
VAS 6. Month	All Patients	2 (± 1.3)	0.9 (± 0.9)	** < 0.001**
	Scrotal	1.9 (± 1.4)	1.1 (± 1)	0.08
	Recurrent	2.1 (± 1.2)	0.7 (± 0.8)	** < 0.001**

*VAS* visual analog scale, All Patients = Scrotal + Recurrent hernia patients, Significance level is p=0.05

## Discussion

In our analysis, radiological assessments conducted at both 1 month and 6 months postoperatively demonstrated comparable and minimal movement of the mesh in both study groups. Notably, there was no further mesh displacement observed in the NFX group. In scrotal hernias and recurrent hernias, which we considered difficult cases, the non-fixation of the mesh did not cause any difference in mesh displacement. Furthermore, whether mesh fixation was used or not did not affect the early recurrence rate because a minimum overlap of 3–4 cm should be maintained at all potential hernia sites.

Although recurrence rates are similar between laparoscopic and open hernia repair, laparoscopic repairs are gaining prominence in inguinal hernia because of the advantages of lower pain and lower wound site infections [12, 13]. The eTEP technique, one of the laparoscopic repairs, is becoming increasingly popular today. In our study, difficult cases such as scrotal and recurrent hernias with a high probability of accidental pneumoperitoneum were included, and the eTEP technique was preferred because it can easily tolerate pneumoperitoneum.

In this study, the demographic characteristics of the patients in both groups were comparable, indicating that the patient population was homogeneous. The occurrence of complications, including seroma, hematoma, and surgical site infection, was comparable between the two groups. In both groups, seroma and hematoma did not require any intervention and were absorbed for 2 months. In our study, we observed that fixation or not in laparoscopic repair had no effect on seroma, hematoma, and wound infection (Table 1).

Some studies have shown that mesh fixation prolongs the operation time [8, 14–16], but some studies have shown that it does not prolong the operation time [9, 17]. In our study, mesh fixation did not prolong the operation time. After appropriate and adequate dissection, we think that it is easy and fast to apply three tackers to the areas where tacker placement will be performed, thanks to the wide view of the eTEP technique.

As no significant complications—such as organ or vascular injuries—could prolong hospitalization in any patient, both groups experienced a standard 1-day hospital stay. Although the hospital stay was 2 days or more in some eTEP studies [18, 19], in our study, whether fixation was performed or not did not affect the length of stay.

In his article describing the eTEP technique, J. Daes stated that accidental pneumoperitoneum occurred often [3]. Pneumoperitoneum was also frequent in our study (Table 1). In scrotal hernias, the peritoneum was opened, and the hernia sac was transected after the hernia sac contents were found to be safe. There was also a high rate of pneumoperitoneum in recurrent hernias. Mesh fixation or not did not affect pneumoperitoneum.

Factors leading to recurrence include inadequate dissection, inadequate mesh size, inadequate overlap of hernia defects with the mesh, folding or rotation of the mesh, and missed hernias [5]. A mesh size of at least 15 × 10 cm is one of the factors that reduce recurrence [20]. Technical problems such as mesh migration and inadequate fixation have been shown to cause recurrences after laparoscopic inguinal hernia repairs [21]. Several meta-analyses have indicated that the absence of mesh fixation does not significantly elevate the risk of recurrence [7, 22]. Upon reviewing the findings of this study, we believe that several factors contribute to the absence of recurrence observed within 6 months in both groups, which are as follows. A single experienced hernia surgeon performed appropriate and adequate dissection in all patients. The mesh size was 15 × 10 cm with at least 3–4 cm overlap over potential hernia areas. Femoral hernia and cord lipoma were not missed. The necessary steps to develop a CV-MPO have been successfully completed. Care was taken not to fold the mesh during desufflation. In our study, it was observed that without mesh fixation did not increase the recurrence rate.

Laparoscopic techniques for inguinal hernia repair are known to be clearly superior to open techniques in return to normal life and work [1]. In a meta-analysis evaluating return to normal life, the absence of mesh fixation in laparoscopic techniques shortened the days of return to daily life [22]. In our study, return to normal life was shorter in the NFX group compared to the FX group.

The debate regarding whether the mesh utilized in laparoscopic inguinal hernia repairs should be fixed remains a contentious topic in the surgical field today. Mesh migration, inadequate mesh size, and inadequate fixation have been mentioned as causes of recurrence after laparoscopic inguinal hernia repair but not as definitive evidence [21, 23]. In randomized studies that marked the mesh with clips and measured mesh displacement without fixation, no recurrence was seen in any patient in the early period [8, 14, 24, 25]. In the study by Yildirim et al., two recurrences were seen in the group without mesh fixation at 6-month follow-up, but there is no evidence that the recurrence occurred due to mesh migration [8]. Studies measuring displacement without mesh fixation have focused on the TEP technique. In studies by Yilmaz et al. on both bilateral and unilateral inguinal hernia repair without mesh fixation, no recurrence cases were observed in both techniques. They also found that mesh displacement in both bilateral and unilateral inguinal hernia repairs was minimal and similar for both techniques [25, 26]. Except for the studies by Yılmaz et al. comparing the eTEP and TEP techniques, there is no mesh displacement study evaluating mesh fixation in the eTEP technique in the literature. We did not find any study other than our study in the literature evaluating mesh fixation in the eTEP technique in a patient population with scrotal and recurrent hernias. In our study, mesh displacement at 6-month follow-up was similar in those with and without fixation (Table 2). In subgroup analysis, total mesh displacement in both scrotal hernias and recurrent hernias was similar in those with and without fixation (Table 3). No more mesh displacement was seen in the NFX group. It is generally believed that the mesh exhibits greater mobility medial to the extraperitoneal region as a result of the eTEP technique; however, our study did not observe any clip movements that would definitively validate this assertion (Table 4).

Mesh fixation creates a thought in the surgeon that it will prevent the mesh from shrinking. Thanks to the nitinol coating that prevents the mesh from shrinking and provides tension, no mesh displacement was observed after 6 months [27]. A study evaluating mesh shrinkage with mesh fixation found that the mesh gradually shrunk at 12 months [28]. In our study, more mesh displacement was observed in both groups at 6 months compared to month 1 (Table 5). At 6 months, this higher mesh displacement can be considered as the presence of mesh shrinkage.

**Fig. 4:**
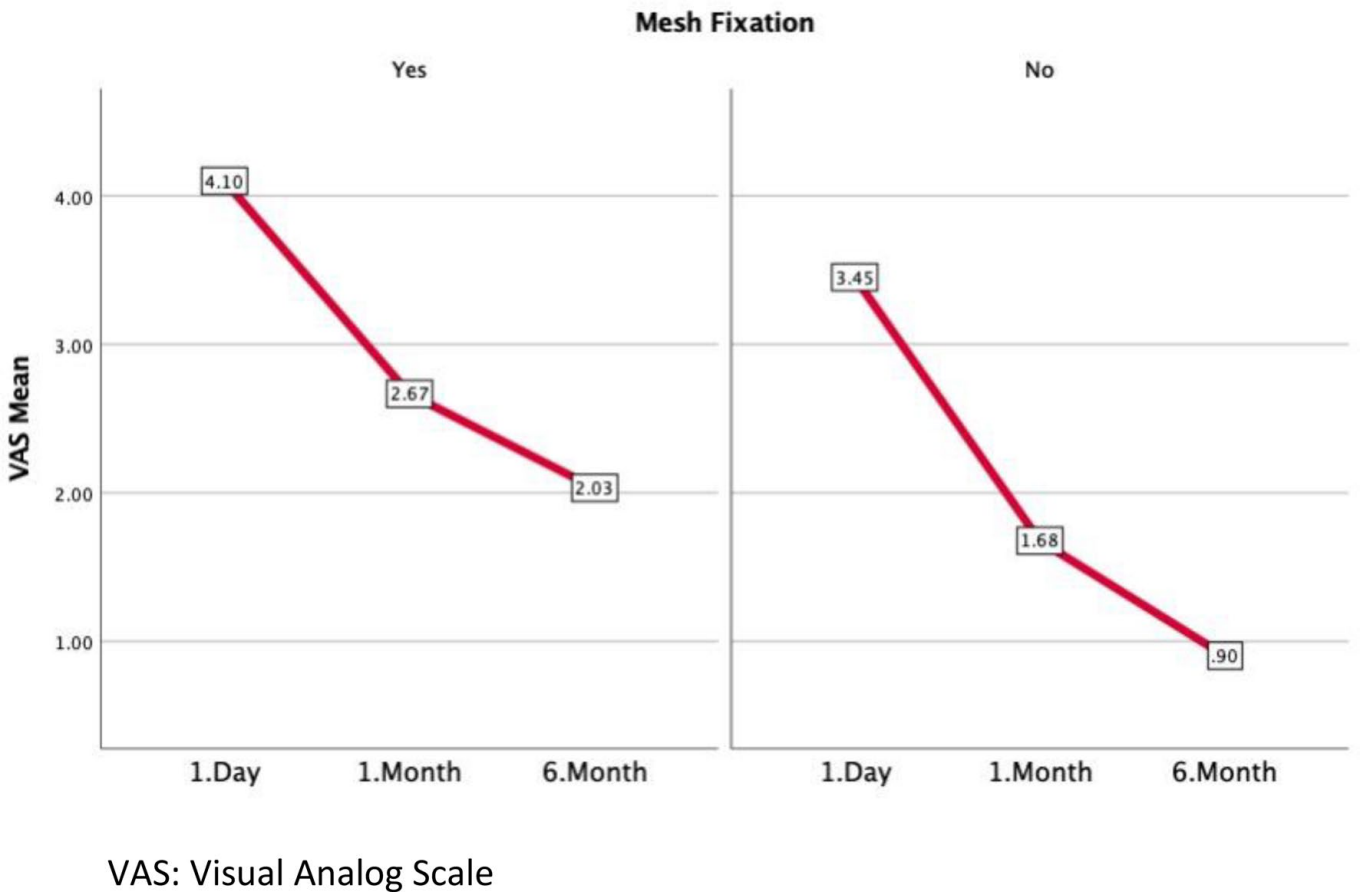
Change of VAS score. VAS: Visual Analog Scale

In an ideal inguinal hernia operation, the surgeon wants to ensure that there is no recurrence and no acute or chronic pain. Although mesh fixation may reassure the surgeon, fixation may lead to an increased risk of acute and chronic pain [29]. Some meta-analyses have found less pain at postoperative day 1 and 6 months in those without mesh fixation [30, 31]. In our study, the pain score was higher in the FX group (Table 6). In both groups, pain scores decreased as time progressed (Fig. 4). The fact that the pain was more in the FX group was thought to be a foreign body and a response of the organism against the foreign body. Although tacker application sites are safe regarding vascular and nerve injury, the body may perceive mesh fixation as trauma.

The most significant limitation of the study is that the follow-up period for recurrence lasted only 6 months. We can consider recurrence during this follow-up period as early recurrence, similar to studies in the literature. Claus et al. were the first to evaluate mesh displacement with quantitative measurements, specifically considering the initial 1-month period in their assessments and evaluating recurrence during this time [14, 24]. Later, Yildirim et al. examined the 6 th postoperative month to calculate recurrence and mesh displacement [8]. Wantanobe et al. conducted a 12-month follow-up and demonstrated that the mesh shrank [26]. We believe that there may not be an optimal postoperative follow-up period to indicate mesh displacement or shrinkage. As time progresses, although the mesh appears to remain in place, it may be shrinking due to its structural nature. We view this uncertainty as another limitation of the study. Based on these considerations, a 6-month follow-up period was implemented in our study because we believe that hernia recurrence resulting from mesh displacement, slippage, or folding may occur in the early follow-up stages.

## Conclusion

The absence of mesh fixation in the eTEP technique does not appear to correlate with an increased risk of mesh displacement or hernia recurrence. In difficult cases, such as scrotal and recurrent hernias, the eTEP technique allows for the safe omission of mesh fixation, which can reduce postoperative pain and allow for a quicker return to normal activities.
